# Dyslipidemia at diagnosis of childhood acute lymphoblastic leukemia

**DOI:** 10.1371/journal.pone.0231209

**Published:** 2020-04-06

**Authors:** Pernille Rudebeck Mogensen, Kathrine Grell, Kjeld Schmiegelow, Ulrik Malthe Overgaard, Benjamin Ole Wolthers, Signe Sloth Mogensen, Allan Vaag, Thomas Leth Frandsen

**Affiliations:** 1 Department of Diabetes and Bone-metabolic Research Unit, University Hospital Rigshospitalet, Copenhagen, Denmark; 2 Department of Pediatrics and Adolescent Medicine, University Hospital Rigshospitalet, Copenhagen, Denmark; 3 Section of Biostatistics, Department of Public Health, University of Copenhagen, Copenhagen, Denmark; 4 Institute of Clinical Medicine, University of Copenhagen, Denmark; 5 Department of Hematology, University Hospital Rigshospitalet, Copenhagen, Denmark; 6 Steno Diabetes Center Copenhagen, Copenhagen, Denmark; German Cancer Research Center (DKFZ), GERMANY

## Abstract

As survival of acute lymphoblastic leukemia (ALL) exceeds 90%, limiting therapy-related toxicity has become a key challenge. Cardio-metabolic dysfunction is a challenge during and after childhood ALL therapy. In a single center study, we measured triglycerides (TG), total cholesterol (TC), high (HDL) and low density lipoproteins (LDL) levels at diagnosis and assessed the association with BMI, early therapy response, on-therapy hyperlipidemia and the toxicities; thromboembolism, osteonecrosis and pancreatitis. We included 127 children (1.0–17.9 years) all treated according to the NOPHO ALL2008 protocol. Dyslipidemia was identified at ALL-diagnosis in 99% of the patients, dominated by reduced HDL levels (98%) and mild hypertriglyceridemia (61%). Hypertriglyceridemia was not associated with body mass index (P = 0.71). Five percent of patients had mild hypercholesterolemia, 14% had mild hypocholesterolemia, 13% had decreased and 1% elevated LDL-levels. Increased TG and TC levels at ALL-diagnosis were not associated with any on-therapy lipid levels. Lipid levels and BMI were not associated to MRD after induction therapy; However, BMI and hypercholesterolemia were associated with worse risk group stratification (P<0.045 for all). The cumulative incidence of thromboembolism was increased both for patients with hypo- (20.0%) and hypercholesterolemia (16.7%) compared to patients with normal TC levels (2.2%) at diagnosis (P = 0.0074). In conclusion, dyslipidemic changes were present prior to ALL-therapy in children with ALL but did not seem to affect dysmetabolic traits during therapy and were not predictive of on-therapy toxicities apart from an association between dyscholesterolemia at time of ALL-diagnosis and risk of thromboembolism. However, the latter should be interpreted with caution due to low number in the groups.

## Introduction

Intensification of therapy has yielded survival rates above 90 percent in children with acute lymphoblastic leukemia (ALL)[1]. However, this intensification has led to severe therapy-related toxicity as well. Thus, more than 50% of children diagnosed with ALL experience toxicity during or after therapy [2,3]. While mechanisms between anti-leukemic drugs and toxicity have been well-studied, studies on potential predictive risk factors prior to ALL therapy have been limited. Identification of such risk modifiers could enable interventions and decrease acute and chronic toxicities in children with ALL.

Obesity and metabolic dysfunction play a major role in survivors of childhood ALL leading to increased risk of morbidity and mortality throughout their life[3–6]. Altered lipid metabolism caused by intensive therapy has been addressed [7–11] and contradictory associations to toxicities such as thromboembolism[12–15], osteonecrosis[16,17] and pancreatitis[18–24] have been reported. Furthermore, abnormal lipid levels prior to ALL therapy[25–29] and obesity at time of ALL-diagnosis have been associated with reduced efficacy of induction therapy, impaired event-free and overall survival[30–32] as well as suggested as a part of the leukomogenisis[33]. However, the mechanism of an altered lipid metabolism in childhood ALL has yet to be elucidated.

Accordingly, the aim of this retrospective study was to explore lipid profiles in children diagnosed with ALL prior to onset of therapy, and its potential association with BMI, early therapy response, on-therapy hyperlipidemia and/or steroid- and asparaginase-associated toxicities; thromboembolism, osteonecrosis and pancreatitis.

## Methods

### Patient population

All children (1 to 17.9 years of age) diagnosed with Philadelphia chromosome negative B-cell precursor or T-lineage ALL from July 2008 to December 2016 at Copenhagen University Hospital Rigshospitalet (Denmark) and enrolled in the Nordic Society of Pediatric Hematology Oncology 2008 (NOPHO ALL2008) protocol were included in the study. Patients with Down syndrome or who received glucocorticoid treatment prior to ALL-diagnosis were excluded from the study cohort.

### NOPHO ALL2008 protocol

The NOPHO ALL2008 protocol is described in detail elsewhere[34]. In brief, patients were stratified into high risk (HR) or non-HR treatment groups at time of diagnosis based on white blood cell count (WBC) and immunophenotype. Following induction (day 29) and consolidation (day 79) patients were subsequently stratified into three risk groups; standard risk (SR), intermediate risk (IR), and HR based on cytogenetics as well as treatment response measured through levels of minimal residual disease (MRD). Induction therapy consisted of a systemic three-drug regimen including Vincristine and Doxorubicine and either Prednisolone or Dexamethasone for non-HR and HR, respectively. Additionally, intrathecal methotrexate or triple therapy was administered depending on CNS involvement (triple for CNS 2/3). Pegylated asparaginase, 6-mercaptopurine and high-dose methotrexate therapy were given from early consolidation phase.

All patients included in the protocol were prospectively registered regarding demographics, anthropometric measurements, and disease characteristics (immunophenotype, WBC, mediastinal mass, MRD, risk group at diagnosis/day 29/day 79, treatment-related toxicities, relapse, secondary malignancy and death). We retrieved data on thromboembolism, osteonecrosis and pancreatitis. Each toxicity was identified based on the Ponte di Legno criteria (PdL)[2]. Venous and/or arterial thromboembolism was registered if confirmed by imaging (ultrasound, CT, or MRI) or by autopsy[2]. Osteonecrosis was indicated by pain in at least one joint, limited activities of daily living and confirmed by MRI [2]. Pancreatitis was registered if at least two of three features were fulfilled: abdominal pain suggestive of pancreatitis; serum lipase or amylase three or more times above upper normal limit (UNL); and characteristic image findings suggestive of pancreatitis (ultrasound, CT, or MRI)[2].

### Blood samples at time of ALL-diagnosis

Blood samples were systematically collected at time of diagnosis before onset of induction therapy prior to or during anesthesia. Patients were fasting at the time point when the blood sample was taken; allowing clear liquids up to two hours and solid foods up to six hours prior to the onset of anesthesia. The lipid analysis (TG, TC, LDL, HDL) were either performed the same day or stored in minus 80 degrees Celsius until batch analysis.

Standardized measurements (WBC, hemoglobin levels, platelet levels, liver biomarkers etc.) were collected before onset of therapy and were extracted from the NOPHO register and medical charts.

### Lipid profiling

The lipid levels were categorized according to the normal limits (lower normal limit [LNL] = 2.5 percentile and UNL = 97.5 percentile) in sex and age matched healthy references defined by an expert panel on integrated guidelines in the Danish Society of Clinical Biocehmistry[35–37] (S1 Table). Dyslipidemia was defined as levels above or below the normal range in one or more of the lipids. Hypertriglyceridemia and hypercholesterolemia, were defined and graded according to the PdL consensus criteria based on triglycerides/cholesterol blood concentrations: mild hypertriglyceridemia/hypercholesterolemia 1–10×UNL, moderate hypertriglyceridemia/hypercholesterolemia 10–20×UNL, severe hypertriglyceridemia/hypercholesterolemia above 20×UNL[2]. Moreover, we defined the lipid levels as normal if within LNL and UNL and decreased if below LNL.

### On-therapy lipid levels

Lipid levels during therapy, including triglyceride and cholesterol levels, were collected from electronic medical charts. Lipids during therapy were not measured systematically. If more than one measurement was available from the same day, the mean of these measurements were calculated.

### Statistics

Age at diagnosis was divided into two groups; children aged 1 to 9.9 years and adolescents 10 to 17.9 years[38]. Body mass index (BMI) was calculated as weight in kilograms divided by height in meters squared and converted to z-scores according to Danish references based on the LMS method[39]. BMI z-scores at diagnosis were divided in three BMI groups defined as: lean body weight <90^th^ percentile, overweight 90^th^–99^th^ percentile, and obesity ≥99^th^ percentile[39]. Triglyceride and cholesterol standard deviation (SD) scores were calculated based on log-transformed values because especially triglycerides are right-skewed. The mean values and SDs for log-triglycerides and log-cholesterol were calculated from the 2.5 and 97.5 percentiles from the distribution of the healthy references assuming that these log-transformed would be approximately normal. As for the BMI z-scores, the triglyceride and cholesterol SD scores reflect how much a (log-) measurement deviates from the average of an age- and sex-matched healthy reference group.

Associations between hypertriglyceridemia/hyper- and hypocholesterolemia/decreased LDL and categorical and continuous characteristics at time of ALL-diagnosis, day 29, or day 79 after diagnosis were analyzed by Fisher’s exact test and Wilcoxon two-sample rank sum test, respectively. Correlations between lipid measurements at diagnosis were evaluated by Spearman’s correlation coefficient.

To illustrate the data, on-therapy mean TG and mean TC were estimated with a cubic smoothing spline with the smoothing parameter chosen by the leave-one-subject-out cross-validation procedure. Subject uniform weights were used in the estimation assigning each patient a weight depending on the patient’s number of repeated measurements. Approximate 95% percentile bootstrap pointwise confidence intervals for the estimated mean curves were obtained from 1000 resampling-subject bootstrap samples. Mean curves were fitted separately for patients with normal/hypertriglyceridemia and normal/hypocholesterolemia (hypercholesterolemia excluded because of too few patients) at time of ALL-diagnosis, respectively, and both TG and TC were log2-transformed.

The 2.5-year cumulative incidences of thromboembolism, osteonecrosis, and pancreatitis were estimated by the Aalen-Johansen estimator considering relapse, secondary malignancy, and death as competing events, and the estimates were compared with Gray’s test. The Cox proportional hazards model was used to calculate age-adjusted hazard ratios of thromboembolism, osteonecrosis, and pancreatitis for the lipid levels at diagnosis with the significance evaluated by Wald tests.

In all analyses two-sided P-values <0.05 were considered statistically significant. All analyses were carried out using the statistical software SAS® version 9.4[40] and R® version 3.5.0[41].

### Ethics

The study has been approved by the ethical institutional review board of The Capital Region of Denmark (Protocol no.: H-2-2010-002).

## Results

A total of 127 children diagnosed with ALL (60% males) with a median age of 4.7 years of age (interquartile range [IQR] 2.9–10.4) fulfilled the inclusion criteria. Information on demographic and disease specific characteristics is presented in Tables 1 and S2. Lipid levels measured at diagnosis were available for 112 patients (88.2%). The 15 patients without available lipid measures did not differ from the included cohort in demographic and disease characteristics. Of the 112 included patients, 82 patients had at least one on-therapy TG measurement and 71 had at least one on-therapy TC measurement.

**Table 1:** Characteristics for the total cohort, triglyceride and total cholesterol groups.

Total cohort		Triglyceride levels at time of ALL-diagnosis N = 112			Total cholesterol levels at time of ALL-diagnosis N = 112				
	N = 127 N (%)	Normal triglyceride levels N = 47 N (%)	Hypertriglyceridemia N = 65 N (%)	P-value	Normal total cholesterol levels N = 91 N (%)	Hypocholesterolemia N = 15 N (%)	P-value (comparison between normal and hypocholesterolemia)	Hypercholesterolemia N = 6 N (%)	P-value (comparison between normal and hypercholesterolemia)
**Variables at time of ALL-diagnosis**									
**Age group**				0.045			0.0017		>0.99
Children, Age <10 years	95 (75)	31 (66)	54 (83)		74 (81)	6 (40)		5 (83)	
Adolescents, Age ≥10 years	32 (25)	16(34)	11 (17)		17 (19)	9 (60)		1 (17)	
**Sex**				0.25			0.78		0.035
Male	76 (60)	30 (64)	34 ((52)		49 (54)	9 (60)		6 (100)	
Female	51 (40)	17 (36)	31 (48)		42 (46)	6 (40)		0 (0)	
**BMI group**				0.71			>0.99		0.68
Lean (<90 percentile)	105 (83)	40 (85)	53 (82)		74 (81)	13 (87)		6 (100)	
Overweight (≥90<99 percentile)	17 (13)	5 (11)	10 (15)		13 (14)	2 (13)		0 (0)	
Obese (≥99 percentile)	5 (4)	2 (4)	2 (3)		4 (5)	0 (0)		0 (0)	
**Risk group at diagnosis**				0.65			0.30		0.0026
Non-high risk	100 (89)	38(80)	50 (77)		73 (80)	14 (93)		1 (17)	
High risk	27 (21)	9 (20)	15 (23)		18 (20)	1 (7)		5 (83)	
**Immunophenotype**				>0.99			>0.99		0.00017
Pre B-cell precursor	110 (87)	41 (87)	56 (86)		82 (90)	14 (93)		1 (17)	
T-cell	17 (13)	6 (13)	9 (14)		9 (10)	1 (7)		5 (83)	
**WBC group**				0.56			0.19		0.069
<50 ×10^9^/L	93 (73)	37 (79)	46 (71)		69 (76)	11 (73)		3 (50)	
50–100 ×10^9^/L	14 (11)	3 (6)	8 (12)		6 (7)	3 (20)		2 (33)	
≥100 ×10^9^/L	20 (16)	7 (15)	11 (17)		16 (17)	1 (7)		1 (17)	
**Mediastinal mass**				0.24			>0.99		0.061
No	102 (94)	43 (98)	59 (91)		84 (94)	14 (100)		4 (67)	
Yes	7 (6)	1 (2)	6 (9)		5 (6)	0 (0)		2 (33)	
Missing	18	3	0		2	1		0	
**Thrombocytes (10^9^/L)^**		54 (20−128)	32 (15−87.5)	0.086	40 (16–93)	30 (14–91)	0.74	69 (30–172)	0.28
**Leukocytes (10^9^/L)^**		6.7 (3.1−24.9)	9.0 (5.3−49.0)	0.13	8.3 (3.5−38.8)	8.1 (3.9−30.0)	0.69	44.7 (5.6−71.2)	0.18
**Hemoglobin (mmol/L)^**		4.2 (3.0−4.9)	4.4 (3.3−5.4)	0.13	4.2 (3.1–5.2)	4.5 (3.1–6.5)	0.37	5.0 (4.3–5.2)	0.36
**C-reactive protein (mg/L)^**		13 (3−33)	10 (5−26)	0.58	9 (3–29)	16 (10–39)	0.092	21 (13–32)	0.14
**Ferritin (μg/L)^**		394 (275−560)	257 (178−362)	0.0015	287 (185–442)	393 (264–925)	0.031	300 (218–427)	0.92
**Sedimentation reaction (mm)^**		74 (42−107)	51 (24−80)	0.038	54 (32–103)	57 (25–105)	0.67	50 (24–140)	0.90
**ALAT (U/L)^**		15 (11−49)	21 (14−38)	0.30	19 (13–41)	21 (14–38)	0.57	15 (7–70)	0.54
**Bilirubin (μmol/L)^**		5 (3−7)	5 (3−8)	0.87	4 (3–7)	7 (5–10)	0.0081	6 (4–10)	0.25
**Early treatment response**									
**Risk group end of induction**				0.24			>0.99		0.024
Standard risk	61 (47)	23 (49)	29 (45)		45 (50)	7 (47)		0 (0)	
Intermediate risk	50 (40)	21 (45)	24 (38)		34 (38)	6 (40)		5 (83)	
High risk	15 (13)	3 (6)	11 (17)		11 (12)	2 (13)		1 (17)	
Missing (dead)	1	0	1		1	0		0	
**MRD at end of induction**				0.21			0.78		>0.99
No	75 (69)	35 (76)	40 (65)		61 (70)	10 (67)		4 (67)	
Yes	33 (31)	11 (24)	22 (35)		26 (30)	5 (33)		2 (33)	
Missing	5	1	3		4	0		0	
**Final risk group (Day 79)**				0.098			0.86		0.025
Standard risk	59 (45)	23 (49)	29 (46)		45 (51)	7 (47)		0 (0)	
Intermediate risk	47(38)	21 (45)	21 (33)		32 (36)	5 (33)		5 (83)	
High risk	19 (16)	3 (6)	13 (21)		12 (13)	3 (20)		1 (17)	
Missing (dead)	2	0	2		2	0		0	
**MRD at day 79**				0.50			0.27		>0.99
No	106 (98)	42 (100)	51 (96)		76 (99)	12 (92)		5 (100)	
Yes	2 (2)	0 (0)	2 (4)		1(1)	1 (8)		0 (0)	
Missing	19	5	12		14	2		1	

P-values are from Fisher’s exact test. Abbreviations: BMI, body mass index; MRD, minimal residual disease; WBC, white blood cell count.

^Median and interquartile range, P-value from Wilcoxon two sample test.

### Lipid levels and BMI at time of ALL-diagnosis

**Fig 1:**
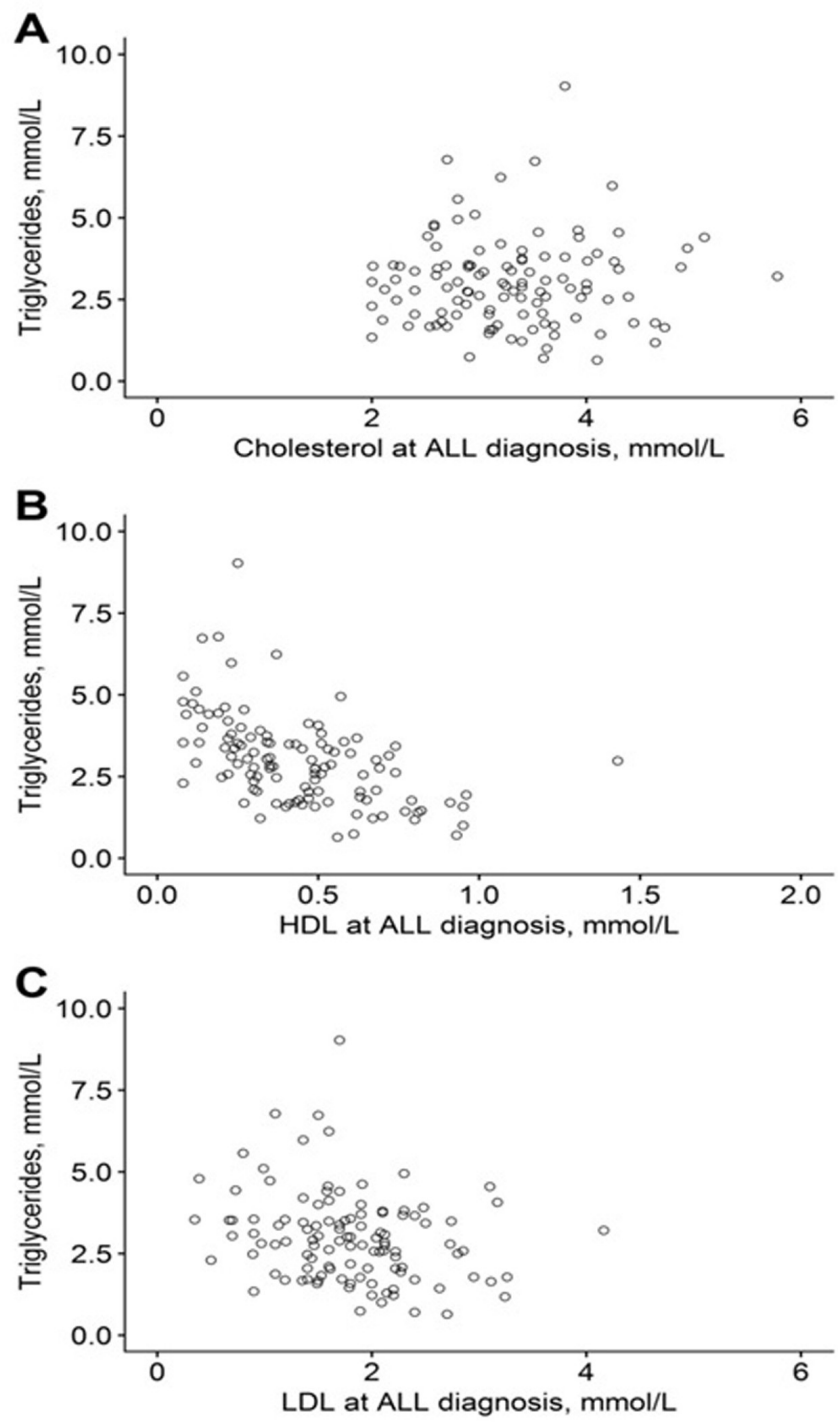
Triglyceride levels and A) total cholesterol levels, B) high-density lipoprotein (HDL) levels, and C) low-density lipoprotein (LDL) levels at time of ALL-diagnosis for 112 patients.

The associations between TG and cholesterol levels at diagnosis are shown in Fig 1A–1C. The inverse correlations between TG and HDL as well as TG and LDL were significant (p<0.0001 and p = 0.0069, respectively), while no correlation between TG and TC was found (p = 0.96).

**Fig 2:**
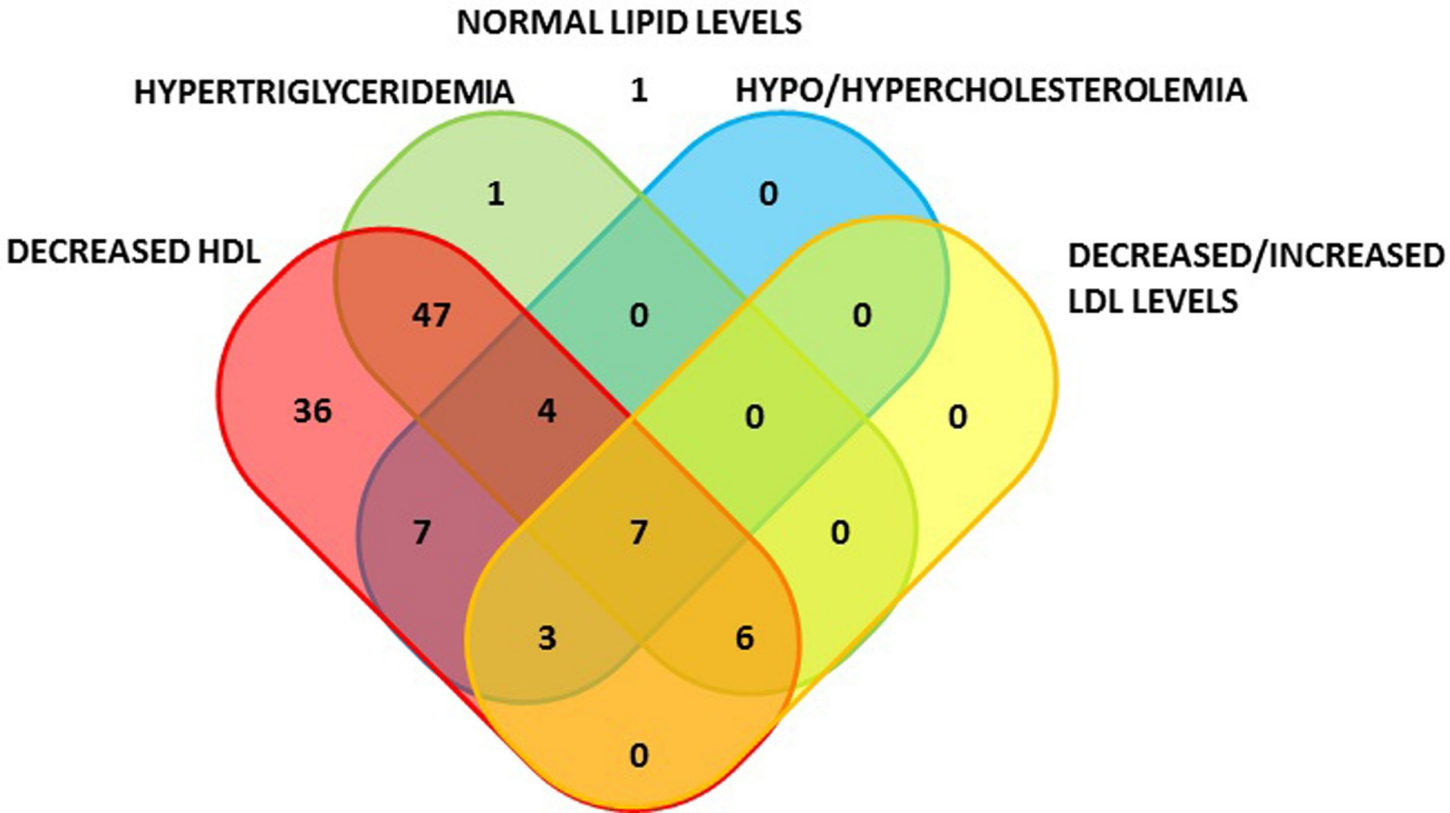
Number of patients with (non)-combined dyslipidemia at time of ALL-diagnosis. One patient did not have dyslipidemia at diagnosis.

Dyslipidemia was identified at time of ALL-diagnosis in 99% (111/112) of patients who had lipid measures at time of ALL-diagnosis (Fig 2). We identified 66% (74/112) of patients with combined dyslipidemia at time of ALL-diagnosis, primarily mild hypertriglyceridemia combined with decreased HDL levels (47/112). HDL levels were reduced in 98% (110/112) and normal in 2% (2/112) and mild hypertriglyceridemia were present in 58% (65/112) of the patients at time of ALL-diagnosis. Mild hypercholesterolemia was present in 5% (6/112), whereof three (3/112) also had hypertriglyceridemia. No patients had moderate or severe hypertriglyceridemia or hypercholesterolemia at time of ALL-diagnosis. Conversely, 14% (15/112) had mild hypocholesterolemia at time of ALL-diagnosis. LDL-levels were normal in 86% of the patients (96/112), and 13% (15/112) had decreased and 1% (1/112) elevated LDL-levels. The combinations of altered lipid levels for the patients are illustrated in Fig 2, and characteristics for patients and TG and TC levels at time of ALL-diagnosis are presented in Table 1, and for LDL and BMI levels in S2 Table. BMI in the study cohort did not differ from healthy sex- and age- matched references (median BMI z-score -0.03 [IQR -0.98−0.82]). No associations were found between mild hypertriglyceridemia/hypercholesterolemia and BMI groups (P = 0.71 and P = 0.68, respectively) at time of diagnosis.

Patients with mild hypertriglyceridemia at time of ALL-diagnosis were significantly younger than patients with normal TG levels (median age 4.2 years [IQR 2.8–7.5] vs. 6.1 years [3.1–12.0], P = 0.043), and the association was also significant when comparing children and adolescents (<10 years vs. ≥10 years, P = 0.045). Contrary, patients with hypocholesterolemia were older than patients with normal TC levels (P = 0.0017) (Table 1).

Immunophenotype and WBC was not associated with mild hypertriglyceridemia (P>0.99 and P = 0.56, respectively) (Table 1). Mediastinal mass was present in six patients with mild hypertriglyceridemia vs. one patient without hypertriglyceridemia, all seven patients were diagnosed with T-lineage leukemia (P = 0.12 and P = 0.24 for patients with T-lineage ALL and for all patients, respectively). Ferritin levels and sedimentation reaction (SR) were significantly lower in patients with hypertriglyceridemia compared to those with normal TG levels (ferritin median 257 [IQR 178‒362] vs. 394 [275‒560]; and SR median 51 [IQR 24‒80] vs. 74 [42‒107]) and no difference were shown for C-reactive protein (CRP) (P = 0.58).

Six patients were present with mild hypercholesterolemia at time of ALL-diagnosis (Table 1). Compared to patients with normal levels of TC, they were characterized by all being males (P = 0.035), five/six had T lineage ALL (P = 0.00017) and thus stratified into HR at time of ALL-diagnosis (P = 0.0026) and six/six stratified into either IR or HR at end of induction and at day 79 (P = 0.024 and P = 0.029, respectively). Furthermore, hypercholesterolemia tended to be associated with tumor burden since four/six had mediastinal mass at time of ALL-diagnosis (P = 0.061) and three of six had WBC count above 50 ×10^9^/L (P = 0.069). Hypercholesterolemia was not statistically associated to BMI group; however, all six patients were lean at time of ALL-diagnosis. Patients with hypocholesterolemia at time of ALL-diagnosis did not differ in characteristics from patients with normal TC levels besides being older (P = 0.0017) and having higher levels of ferritin and bilirubin at time of ALL-diagnosis (P = 0.031 and P = 0.0081, respectively).

### Early treatment response

MRD measures were missing for five patients at the end of induction (EOI) and additional 14 patients at day 79 (Table 1). Patients missing MRD at EOI or day 79 were either stratified into HR already at day 15/29 due to poor response or died during therapy (one patient died during induction and two patients died during remission before day 79. No association was found between lipid levels or BMI at time of ALL-diagnosis and MRD measures at end of induction and/or at day 79. Using risk stratification at the end of induction and at day 79 as a proxy for risk of therapy outcome we found a significant association between hypercholesterolemia at time of ALL-diagnosis and a poorer risk stratification at end of induction and day 79 (P = 0.024 and P = 0.025, respectively). Likewise, overweight/obesity at time of ALL-diagnosis was associated with poorer risk group stratification both after induction (P = 0.049) and at day 79 (P = 0.017) (S2 Table). Hypertriglyceridemia at time of ALL-diagnosis was not associated to risk group stratification at end of induction (P = 0.24) though a tendency were seen at day 79 (P = 0.098) (Table 1).

### On-therapy hypertriglyceridemia and hypercholesterolemia

Of the 112 patients with lipid measurements at time of ALL-diagnosis 82 patients had 1–98 (median 21, total 2070) on-therapy TG measurements, and of these 71 patients had 1–92 (median 23, total 1752) on-therapy TC measurements, reflecting that the sampling of on-therapy lipids was highly irregular. The sampling frequency varied from 1–548 (median 4, IQR 1–8) days between on-therapy TG measurements from the same patient. Most of the lipid measurements were from the first 270 days of therapy (during induction and asparaginase therapy) (91.5% of the TG and 92.5% of the TC measurements).

**Fig 3:**
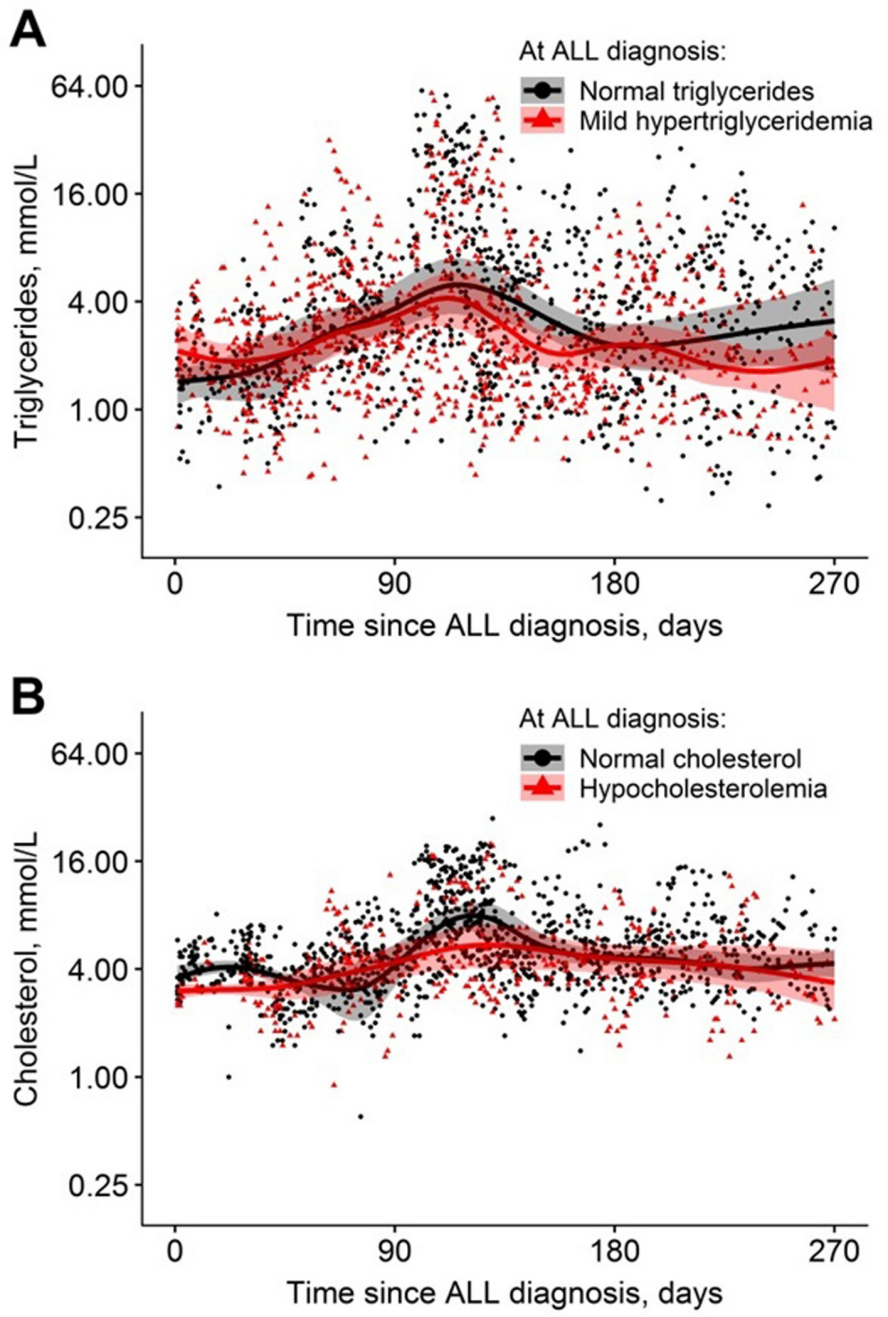
On-therapy levels of A) triglycerides with mean curves according to normal and mild hypertriglyceridemia at time of ALL-diagnosis, and B) total cholesterol according to normal and hypocholesterolemia at time of ALL-diagnosis. The shaded areas show the approximate 95% percentile bootstrap pointwise confidence intervals for the estimated mean curves.

On-therapy TG and total TC levels within the first 270 days of therapy are demonstrated according to levels at time of ALL-diagnosis in Fig 3 for 80 patients with a median of 20 TG measurements (IQR 11–32) and 65 patients (3 patients with hypertriglyceridemia excluded) with median 23 TC measurements (IQR 14–32), respectively. We could not detect a difference in development in TG or TC over time for the groups defined by at-diagnosis lipid levels illustrated by fitted curves with overlapping confidence intervals in Fig 3. The curve for hypercholesterolemia could not be illustrated due to low numbers.

Neither was a difference identified when comparing the fitted curves for TG and TC on-therapy levels for induction therapy groups (prednisolone [non-HR] vs. dexamethasone [HR], respectively) (S1 Fig).

To illustrate the individual variation in TG on therapy, S2 and S3 Figs show the on-therapy TG SD scores and raw measurements, respectively, for the 16 patients with most measurements within the first year of therapy. S4 and S5 Figs similarly show the on-therapy TC SD scores and raw measurements for the same 16 patients.

### Toxicities

The cumulative incidences of thromboembolism, osteonecrosis, and pancreatitis were 5.5% (95% CI 2.4–10.5), 7.2% (3.5–12.7), and 18.2% (12.1–25.4), respectively, and were not associated with age group in the study cohort (P>0.26 for all) (Table 2). The cumulative incidence of any toxicity was 31.1% (22.9–39.4) with no significant association with age group or any of the lipid levels at diagnosis (P>0.11 for all). The cumulative incidence of thromboembolism was significantly higher for patients with hypocholesterolemia (20.0%) and hypercholesterolemia (16.7%) at diagnosis compared to patients with normal TC levels (2.2%) (P = 0.0074). Correspondingly, the age-adjusted hazard ratio of thromboembolism was significantly associated with dyscholesterolemia (9.3, 95% CI 1.7–50.8, P = 0.011) (S3 Table).

**Table 2:** Cumulative incidences of toxicity.

		Thromboembolism		Osteonecrosis		Pancreatitis	
	Total cohort N (%)	2.5-year cumulative incidence, % (95% CI)	P-value	2.5-year cumulative incidence, % (95% CI)	P-value	2.5-year cumulative incidence, % (95% CI)	P-value
**Total**	127	5.5 (2.4–10.5)		7.2 (3.5–12.7)		18.2 (12.1–25.4)	
**Age groups**			0.26		0.55		0.32
Children, Age <10 years	95 (75)	4.2 (1.4–9.7)		6.4 (2.6–12.6)		20.1 (12.7–28.7)	
Adolescents, Age ≥10 years	32 (25)	9.4 (2.3–22.5)		9.8 (2.4–23.6)		12.5 (3.9–26.5)	
**Triglyceride levels at time of ALL-diagnosis**			0.67		0.63		0.32
Normal (<UNL)	47 (42)	6.4 (1.6–15.9)		8.6 (2.7–18.9)		12.8 (5.1–24.0)	
Mild hypertriglyceridemia (≥UNL and <10xUNL)	65 (58)	4.6 (1.2–11.8)		6.3 (2.0–14.2)		20.0 (11.3–30.5)	
**Total cholesterol levels at time of ALL-diagnosis**			0.0074		0.49		0.52
Hypocholesterolemia (<LNL)	15 (14)	20.0 (4.5–43.3)		13.3 (2.0–35.4)		20.0 (4.5–43.3)	
Normal	91 (81)	2.2 (0.004–6.9)		6.7 (2.7–13.3)		17.6 (10.6–26.1)	
Mild hypercholesterolemia (≥UNL and <10xUNL)	6 (5)	16.7 (4.8–54.9)		-		-	
**LDL levels at time of ALL-diagnosis**			0.94		0.59		0.48
Decreased (<LNL)	15 (13)	6.7 (0.4–26.9)		13.3 (2.0–35.5)		26.7 (7.7–50.5)	
Normal	96 (86)	5.2 (1.49–11)		6.4 (2.6–12.7)		15.6 (9.2–23.6)	
Increased (≥UNL)	1 (1)	-		-		-	
**HDL levels at time of ALL-diagnosis**			-		-		-
Normal	2 (2)	-		-		-	
Decreased (<LNL)	110 (98)	5.5 (2.2–10.8)		7.4 (3.4–13.4)		17.3 (10.9–24.9)	
**BMI at time of ALL-diagnosis**			0.83		-		0.51
Lean	105 (83)	4.8 (1.8–10.1)		8.1 (2.8–14.7)		17.4(10.8–25.3)	
Overweight	17 (13)	5.9 (3.4–24.3)		-		17.7(4.1–39.1)	
Obese	5 (4)	-		-		40.0 (3.1–78.6)	

Lipids at time of ALL-diagnosis are available for 112 of the 127 patients. P-values are from Gray’s test. Abbreviations: UNL, upper normal limit; LNL, lower normal limit; CI, confidence interval; LDL, low density lipoprotein; HDL, high density lipoprotein; BMI, body mass index.

No other significant associations were found between the lipid levels or BMI level at time of ALL-diagnosis and age-adjusted hazard rate of thromboembolism (P>0.78) (S3 Table), and no significant associations with osteonecrosis or pancreatitis (P>0.25) (S3 Table).

## Discussion

Tumor development influences the host lipid metabolism in animals by inducing hyperlipidemia partly through reprogramming of hepatic lipoprotein homeostasis[42]. This provides enhanced cholesterol uptake by the tumor and supports its growth[42]. Metabolic dysfunction is likewise presented in cancer patients with cancer-associated cachexia[43]. However, only scarce literature exists on this complex multi organ syndrome in children with ALL. We demonstrated that 99% of the children had dyslipidemia at time of ALL-diagnosis, with abnormalities in at least one type of lipids, highly dominated by decreased HDL levels and hypertriglyceridemia and to less extent by altered TC and LDL levels. This is compatible with previous findings from smaller ALL cohorts[25–28] possibly caused by the cancer-induced catabolism[44]. The catabolism changes substrate supply priorities and stimulated adipose tissue lipolysis in peripheral and visceral adipose storages, as well as a decreased fatty acid oxidation. The increased release of free fatty acids instigate elevated hepatic production of TG and secretion of very low density lipoprotein (VLDL)[10,44]. Furthermore, catabolism leads to decreased levels and compositional changes of plasma LDL and HDL[44].

Overweight/obesity at time of ALL-diagnosis was associated to a poorer risk group stratification in our study indicating a poorer treatment outcome which previously have been shown by other studies[30,31]. Both interpatient variation of pharmacokinetics (over- and under-treatment) as well as protective mechanisms in adipocytes have been suggested to explain a worse therapy outcome in obese patients with ALL[30,45,46]. Furthermore, it has been discussed if obesity takes part in the’ leukemogenesis’ in adults, though it was not supported by our results[33,47,48].

Likewise, hypercholesterolemia at time of ALL-diagnosis was associated to a worse risk group stratification probably explained by immunophenotype since five of six of the patients with hypercholesterolemia had T-cell ALL and will, according to the protocol, receive IR or HR therapy. MRD measures did not support the findings possibly due to lack of power and available MRD measures for HR patients. The lipid levels at diagnosis have not previously been associated to treatment response. However, an association to tumor burden at diagnosis has been indicated[26,27]. Also, differences in the leukomogenesis of T-ALL, compared to B-ALL, might be a part of the association with hypercholesterolemia, but this need to be further determined. We demonstrated a non-significant association between hypercholesterolemia at diagnosis and WBC >50x10^9^/L as well as an association with risk of mediastinal mass, suggesting an association between increased TC and higher tumor burden[43]. Hypercholesterolemia was strongly associated to immunophenotype, which suggests that T-cell leukemia induces a different host-response, with increased levels of TC, than B-cell leukemia. However, these results should be interpreted with care due to low numbers in the group. Furthermore, TC was not associated to any of the measured liver numbers. No literature is available on this topic but needs further research.

The sampling frequency and levels of on-therapy TG and TC varied considerably between patients throughout the first year of therapy, and sampling frequency was typically higher in periods with high levels, making it difficult to classify a patient as having high or low levels. We hypothesized that increased lipid levels at time of ALL-diagnosis could predict on-therapy lipid levels; however, our data did not support this. In fact, patients with elevated TG levels at time of ALL-diagnosis had lower levels compared to those with normal or decreased levels at time of ALL-diagnosis. This probably reflects two different mechanisms for increased TG and TC levels at time of ALL-diagnosis and during asparaginase/dexamethasone therapy. On-therapy lipid levels are known to be related to the intense therapy with glucocorticoids and asparaginase[2,11,49,50], supported by the findings in the present study with the highest levels of TG and TC during therapy with both asparaginase and dexamethasone. The wide variation in TG and TC levels during therapy for the individual patient points to studies using systematic measurements in defined time/therapy intervals to obtain a better understanding of the lipid response during therapy, could be useful to further understand the fluctuations and relationship to toxicities. Glucocorticoids are known to have anabolic side effects in children with ALL and cause weight gain[51]. The combination of anabolism and decreased physical activity will lead to metabolic dysfunction (including obesity, (pre-)diabetes and cardiovascular disease) in children with ALL both during and after treatment[52,53].

Thromboembolism in patients with ALL are usually related to complications with central venous lines[14]. However, our age-adjusted analyses suggests an increased incidence of thromboembolism for both hypo- and hypercholesterolemia at time of ALL-diagnosis, supporting possible changes in the vascular wall exists at time of ALL-diagnosis[44] and may persist during therapy. Although, central venous line and circulating factors or other pro-thrombotic factors may also impact the risk of thromboembolism. Unfortunately, we did not measure VLDL levels in this study, which hypothetically would track with the TG levels[44]. Furthermore, our results are in line with a large randomized controlled trial showing an association between the formation of blood cloths and cholesterol levels, and could indicate a need for statin therapy, or other lipid lowering therapy, for patients being at high risk of thromboembolism without having hyperlipidemia[54]. Rank et al. have shown an association between risk of thromboembolism and immunophenotype as well as mediastinal mass[13] which support our finding on hypercholesterolemia at time of ALL-diagnosis being associated with all three variables. The exact mechanism behind the association between hypocholesterolemia and risk of thromboembolism needs further investigation.

The lipid levels at time of ALL-diagnosis were not associated to osteonecrosis. However, an association between osteonecrosis and on-therapy lipid levels were previously demonstrated by our group in a larger cohort [17]. However, this could support the notion of different causes and implications of elevated TG levels at diagnosis versus during treatment or reflect the low number of patients with ON in the present study. No association between the lipid levels at diagnosis and pancreatitis were shown. Risk of pancreatitis may primarily be caused by the intense asparaginase therapy[55], possibly by asparaginase-induced hypertriglyceridemia; although, this remains to be demonstrated in children with ALL.

This study cohort was treated according to the same protocol and population based, representing all patients below 18 years of age, diagnosed with ALL in the eastern part of Denmark since 2008 (approximately 50% of the national cohort). However, the study is limited by the size of the cohort, its retrospective design and lack of systematic lipid measures during therapy.

Cardio-metabolic conditions are emerging concerns in children during and after ALL therapy. To clarify this major issue a better understanding of the interaction between the dyslipidemic host-response, prior to and during ALL therapy, and the ALL is needed.

## Supporting information

S1 Fig On-therapy levels of A) triglycerides and B) total cholesterol, both with fitted smoothed spline curves according to induction therapy (prednisolone vs. dexamethasone). The shaded areas show the approximate 95% percentile bootstrap pointwise confidence intervals for the estimated mean curves.
(DOCX)

S2 Fig Triglyceride SD scores for 16 patients (4 patients within each Fig A-D) with more than 36 measurements within the first year of therapy.
(DOCX)

S3 Fig On-therapy triglycerides for 16 patients (4 patients within each Fig A-D) with more than 36 measurements within the first year of therapy.
(DOCX)

S4 Fig Total cholesterol SD scores for 16 patients (4 patients within each Fig A-D) with more than 36 measurements within the first year of therapy.
(DOCX)

S5 Fig On-therapy total cholesterol for 16 patients (4 patients within each Fig A-D) with more than 36 measurements within the first year of therapy.
(DOCX)

S1 Table Normal age- and sex adjusted lipid levels determined by an expert panel from the Danish Society of Clinical Biochemistry based on clinical studies^35,36,37^. (DOCX)

S2 Table Characteristics for LDL and BMI groups. (DOCX)

S3 Table Age-adjusted hazard ratios for toxicities. (DOCX)

## Abbreviations

ALL: Acute lymphoblastic leukemia
BMI: Body mass index
CNS: Central nervous system
CT: Computed tomography scan
HDL: High density lipoprotein
HR: High risk
IR: Intermediate risk
LDL: Low density lipoprotein
LNL: Lower normal limit
MRD: Minimal residual disease
MRI: Magnetic Resonance Imaging
NOPHO: Nordic Society of Pediatric Hematology Oncology
SR: Standard risk
TC: Total cholesterol
TG: Triglyceride
UNL: Upper normal limit
WBC: White blood cell
