# Supplementary material for: Dyslipidemia at diagnosis of childhood acute lymphoblastic leukemia
Source: PLoS One. 2020 Apr 6;15(4):e0231209. doi: 10.1371/journal.pone.0231209 (PMC7135240; doi:10.1371/journal.pone.0231209)

**Supplemental Figure S1. On-therapy levels of A) triglycerides and B) total cholesterol, both with fitted smoothed spline curves according to induction therapy (prednisolone vs. dexamethasone). The shaded areas show the approximate 95% percentile bootstrap pointwise confidence intervals for the estimated mean curves.**


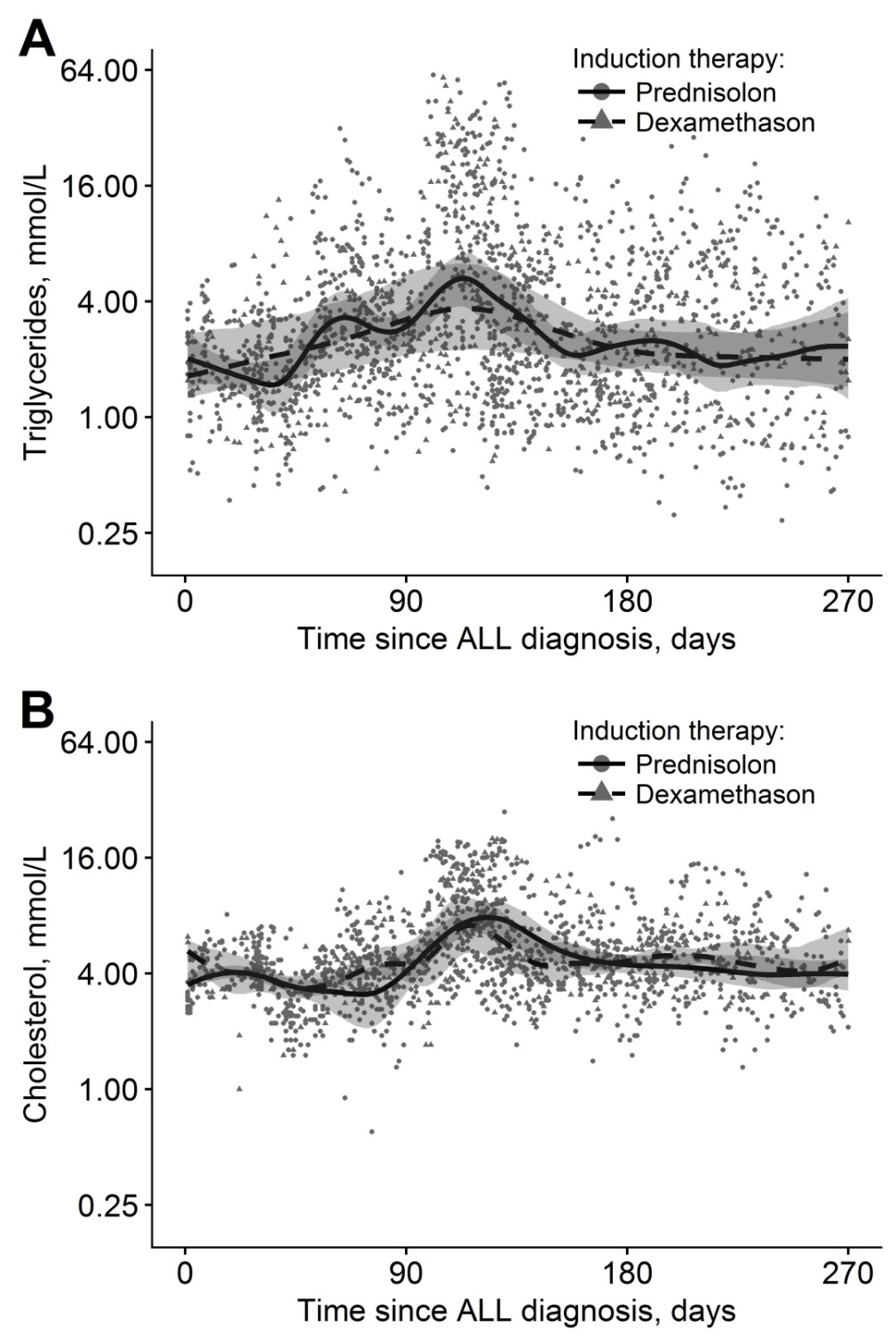

Supplement: S1 Fig — On-therapy levels of A) triglycerides and B) total cholesterol, both with fitted smoothed spline curves according to induction therapy (prednisolone vs. dexamethasone). The shaded areas show the approximate 95% percentile bootstrap pointwise confidence intervals for the estimated mean curves. (DOCX) [file pone.0231209.s001.docx]
