# Supplementary material for: Dyslipidemia at diagnosis of childhood acute lymphoblastic leukemia
Source: PLoS One. 2020 Apr 6;15(4):e0231209. doi: 10.1371/journal.pone.0231209 (PMC7135240; doi:10.1371/journal.pone.0231209)

**Supplemental Figure S3. On-therapy triglycerides for 16 patients (4 patients within each figure A-D) with more than 36 measurements within the first year of therapy.**


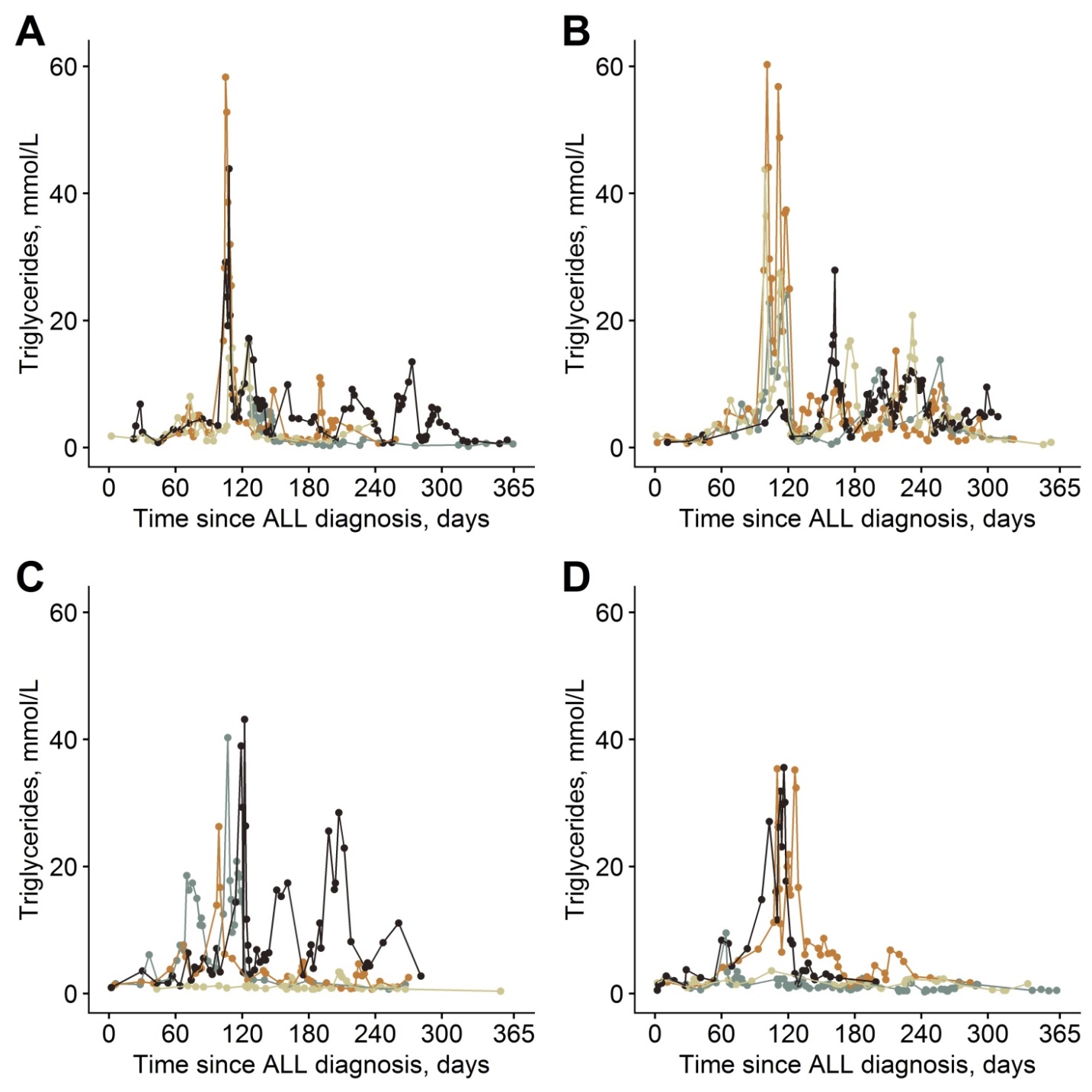

Supplement: S3 Fig — On-therapy triglycerides for 16 patients (4 patients within each Fig A-D) with more than 36 measurements within the first year of therapy. (DOCX) [file pone.0231209.s003.docx]
