# Supplementary material for: Dyslipidemia at diagnosis of childhood acute lymphoblastic leukemia
Source: PLoS One. 2020 Apr 6;15(4):e0231209. doi: 10.1371/journal.pone.0231209 (PMC7135240; doi:10.1371/journal.pone.0231209)
Supplement: S1 Table — (DOCX) [file pone.0231209.s006.docx]

**Supplemental Table S1. Normal age- and sex adjusted lipid levels determined by an expert panel from the Danish Society of Clinical Biochemistry based on clinical studies^35,36,37^.**

| **Lipid** | **Age (years)** | **Levels for both sexes** | **Levels for girls** | **Levels for boys** | **Unit** |
| --- | --- | --- | --- | --- | --- |
| **HDL^36^** | 1 < 4 | 0.8 - 1.6 |  |  | mmol/L |
| **HDL^36^** | 4 < 6 | 0.9 - 1.9 |  |  | mmol/L |
| **HDL^35^** | 6 < 19 |  | 1.0 - 2.3 |  | mmol/L |
| **HDL^35^** | 6 < 14 |  |  | 1.0 - 2.3 | mmol/L |
| **HDL^35^** | 14 < 19 |  |  | 0.8 - 2.0 | mmol/L |
| **LDL^37^** | 1 < 6 |  | 0.69 - 2.55 | 0.83 - 2.81 | mmol/L |
| **LDL^35^** | 6 < 19 | 1.1 - 3.4 |  |  | mmol/L |
| **Total cholesterol^37^** | 1 < 3 |  | 1.9 - 5.1 | 2.8 - 5.7 | mmol/L |
| **Total cholesterol^37^** | 3 < 6 |  | 3.2 - 6.1 | 2.9 - 6.0 | mmol/L |
| **Total cholesterol^35^** | 6 < 19 | 2.7 - 5.5 |  |  | mmol/L |
| **Triglycerides^37^** | 1 < 19 | 0.48 - 2.69 |  |  | mmol/L |
