# Supplementary material for: Dyslipidemia at diagnosis of childhood acute lymphoblastic leukemia
Source: PLoS One. 2020 Apr 6;15(4):e0231209. doi: 10.1371/journal.pone.0231209 (PMC7135240; doi:10.1371/journal.pone.0231209)
Supplement: S2 Table — (DOCX) [file pone.0231209.s007.docx]

**Supplemental Table S2. Characteristics for LDL and BMI groups.**

|  |  | **Low density lipoprotein levels at diagnosis**  **N=111** | | | |  | | **Body Mass Index**  **N=127** | | |  |
| --- | --- | --- | --- | --- | --- | --- | --- | --- | --- | --- | --- |
|  |  | **Normal LDL levels at ALL diagnosis**  **N=96**  **N (%)** | **Decreased LDL levels**  **at ALL diagnosis**  **N=15**  **N (%)** | **P-value** | |  | | **Lean**  **(BMI< 90^th^ percentile)**  **N=105**  **N (%)** | **Overweight/Obese**  **(BMI≥ 90^th^ percentile)**  **N=22**  **N (%)** | **P-value** |  |
|  | **Variables at ALL diagnosis** | | | | | | | | | |  |
|  | **Age group** |  |  | 0.75 |  | |  | |  | 0.10 |  |
|  | Children, Age <10 years | 74 (77) | 11 (73) |  |  | | 82 (78) | | 13 (59) |  |  |
|  | Adolescents, Age ≥10 years | 22 (23) | 4 (27) |  |  | | 23 (22) | | 9 (41) |  |  |
|  |  |  |  |  |  | |  | |  |  |  |
|  | **Sex** |  |  | 0.42 |  | |  | |  | 0.48 |  |
|  | Male | 56 (58) | 7 (47) |  |  | | 61 (58) | | 15(68) |  |  |
|  | Female | 40 (42) | 8 (53) |  |  | | 44 (42) | | 7 (32) |  |  |
|  |  |  |  |  |  | |  | |  |  |  |
|  | **BMI group** |  |  | 0.83 |  | |  | |  | - |  |
|  | Lean (<90 percentile) | 78 (81) | 14 (93) |  |  | | - | | - |  |  |
|  | Overweight  (≥90<99 percentile) | 14 (15) | 1 (7) |  |  | | - | | - |  |  |
|  | Obese  (≥99 percentile) | 4 (4) | 0 (0) |  |  | | - | | - |  |  |
|  |  |  |  |  |  | |  | |  |  |  |
|  | **Risk group at diagnosis** |  |  | 0.73 |  | |  | |  | 0.57 |  |
|  | Non-high risk | 75 (78) | 13 (87) |  |  | | 84 (80) | | 16 (73) |  |  |
|  | High risk | 21 (22) | 2 (13) |  |  | | 21 (20) | | 6 (27) |  |  |
|  |  |  |  |  |  | |  | |  |  |  |
|  | **Immunophenotype** |  |  | 0.21 |  | |  | |  | 0.49 |  |
|  | Pre B-cell precursor | 82 (85) | 15 (100) |  |  | | 92 (88) | | 18 (82) |  |  |
|  | T-cell | 14 (15) | 0 (0) |  |  | | 13 (12) | | 4 (18) |  |  |
|  |  |  |  |  |  | |  | |  |  |  |
|  | **Tumor burden** |  |  |  |  | |  | |  |  |  |
|  | **WBC group** |  |  | 0.79 |  | |  | |  | 0.55 |  |
|  | <50 ×10^9^/L | 72 (75) | 11 (74) |  |  | | 78 (61) | | 15 (68) |  |  |
|  | 50–100 ×10^9^/L | 8 (8) | 2 (13) |  |  | | 12 (11) | | 2 (9) |  |  |
|  | ≥100 ×10^9^/L | 16 (17) | 2 (13) |  |  | | 15 (14) | | 5 (23) |  |  |
|  | **Mediastinal mass** |  |  | 0.59 |  | |  | |  | 0.20 |  |
|  | No | 87 (93) | 14 (100) |  |  | | 95 (77) | | 19 (86) |  |  |
|  | Yes | 7 (7) | 0 (0) |  |  | | 6 (6) | | 3 (14) |  |  |
|  | Missing | 2 | 1 |  |  | | 4 | | 0 |  |  |
|  |  |  |  |  |  | |  | |  |  |  |
|  | Thrombocytes(10^9^/L)^ | 40 (18−96) | 23 (14−111) | 0.67 |  | | 39 (14−91) | | 28 (18−143) | 0.82 |  |
|  | Leukocytes (10^9^/L)^ | 9.0 (3.5−41.6) | 6.3 (4.2−30.0) | 0.81 |  | | 11.4 (3.1−5.2) | | 21.6 (6.7−98.0) | 0.09 |  |
|  | Hemoglobin (mmol/L)^ | 4.3 (3.1−5.2) | 4.3 (3.3−5.1) | 0.94 |  | | 4.3 (3.1 −5.2) | | 4.3 (2.9−5.0) | 0.48 |  |
|  | C-reactive protein (mg/L)^ | 12 (3−31) | 12 (6−18) | 0.52 |  | | 11 (3−31) | | 16 (8−28) | 0.30 |  |
|  | Ferritin (µg/L)^ | 295 (186−455) | 288 (264−819) | 0.31 |  | | 309 (211−466) | | 271 (181−687) | 0.65 |  |
|  | Sedimentation reaction (mm)^ | 55 (34−103) | 55 (25−104) | 0.93 |  | | 55 (28−104) | | 46 (25­−75) | 0.22 |  |
|  | ALAT (U/L)^ | 18 (12−41) | 21 (17−38) | 0.31 |  | | 20 (13−45) | | 25 (14−­54) | 0.60 |  |
|  | Bilirubin (µmol/L)^ | 5 (3−7) | 5 (3−10) | 0.64 |  | | 5 (3−7) | | 6 (5−10 | 0.054 |  |
|  | **Early treatment response** | | | |  | |  | | | |  |
|  | **Risk group end of induction** |  |  | >0.99 |  | |  | |  | 0.049 |  |
|  | Standard risk | 45 (47) | 7 (47) |  |  | | 53 (50) | | 8 (38) |  |  |
|  | Intermediate risk | 39 (41) | 6 (40) |  |  | | 43 (41) | | 7 (33) |  |  |
|  | High risk | 11 (12) | 2 (13) |  |  | | 9 (9) | | 6 (29) |  |  |
|  | **Missing (dead)** | 1 | 0 |  |  | | 0 | | 1 |  |  |
|  |  |  |  |  |  | |  | |  |  |  |
|  | **MRD end of induction** |  |  | 0.77 |  | |  | |  | 0.15 |  |
|  | No | 65 (71) | 10 (67) |  |  | | 77 (73) | | 9 (53) |  |  |
|  | Yes | 27 (29) | 5 (33) |  |  | | 28 (27) | | 8 (47) |  |  |
|  | Missing | 4 | 0 |  |  | | 0 | | 5 |  |  |
|  |  |  |  |  |  | |  | |  |  |  |
|  | **Final risk group (Day 79)** |  |  | 0.69 |  | |  | |  | 0.017 |  |
|  | Standard risk | 45 (48) | 7 (47) |  |  | | 53 (50) | | 8 (40) |  |  |
|  | Intermediate risk | 37 (39) | 5 (33) |  |  | | 42 (40) | | 5 (25) |  |  |
|  | High risk (4 SCT) | 12 (13) | 3 (20) |  |  | | 10 (10) | | 7 (35) |  |  |
|  | **Missing (dead)** | 2 | 0 |  |  | | 0 | | 2 |  |  |
|  |  |  |  |  |  | |  | |  |  |  |
|  | **MRD at day 79** |  |  | 0.26 |  | |  | |  | 0.23 |  |
|  | No | 81 (99) | 12 (92) |  |  | | 94 (99) | | 12 (92) |  |  |
|  | Yes | 1 (1) | 1 (8) |  |  | | 1 (1) | | 1 (8) |  |  |
|  | **Missing** | 14 | 2 |  |  | | 10 | | 9 |  |  |
|  |  |  |  |  |  | |  | |  |  |  |

Values for increased LDL levels and HDL are not shown due to small numbers. P-values are from Fisher’s exact test. Abbreviations: BMI, body mass index; MRD, minimal residual disease; WBC, white blood cell count. ^Median and interquartile range, P-value from Wilcoxon two sample test.
