# Supplementary material for: Dyslipidemia at diagnosis of childhood acute lymphoblastic leukemia
Source: PLoS One. 2020 Apr 6;15(4):e0231209. doi: 10.1371/journal.pone.0231209 (PMC7135240; doi:10.1371/journal.pone.0231209)
Supplement: S3 Table — (DOCX) [file pone.0231209.s008.docx]

**Supplemental Table S3. Age-adjusted hazard ratios for toxicities**

|  |  | **Thromboembolism** |  | **Osteonecrosis** |  | **Pancreatitis** |  |  |
| --- | --- | --- | --- | --- | --- | --- | --- | --- |
|  | **Variables at ALL diagnosis** | **Age-adjusted**  **hazard ratio (95%CI)**  **P-value** | **P-value** | **Age-adjusted**  **hazard ratio (95%CI)**  **P-value** | **P-value** | **Age-adjusted**  **hazard ratio (95%CI)**  **P-value** | **P-value** |  |
|  |  |  |  |  |  |  |  |  |
|  | **Mild hypertriglyceridemia** | 0.81  (0.16−4.23) | 0.81 | 0.85  (0.20−3.52) | 0.82 | 1.49  (0.56−3.37) | 0.42 |  |
|  |  |  |  |  |  |  |  |  |
|  | **Dyscholesterolemia** | 9.84  (1.69−57.28) | 0.011 | 1.16  (0.22−6.11) | 0.86 | 0.96  (0.27−3.37) | 0.95 |  |
|  |  |  |  |  |  |  |  |  |
|  | **Decreased/increased LDL levels** | 1.15  (0.13−9.89) | 0.90 | 1.91  (0.38−9.51) | 0.43 | 1.91  (0.63−5.77) | 0.25 |  |
|  |  |  |  |  |  |  |  |  |
|  | **BMI (overweight/obese)** | 0.74  (0.09−6.24) | 0.78 | -  - | - | 1.56  (0.57−4.25) | 0.38 |  |
|  |  |  |  |  |  |  |  |  |

Hazard ratios are calculated using multiple regressions and compared between children and adolescents with Cox proportional hazard ratios. P-values are calculated from Wald test**.** Decreased/increased cholesterol and LDL levels as well as overweight and obese BMI are combined in one group and compared to normal levels due to low numbers. Age-adjusted hazard ratio for HDL levels for all toxicities and BMI levels for osteonecrosis are not shown due to small numbers.
